# A Detoxification Enzyme for *Apis mellifera* Newly Characterized by Recombinant Expression: 10-Formyl Tetrahydrofolate Dehydrogenase

**DOI:** 10.3389/finsc.2022.829869

**Published:** 2022-03-24

**Authors:** Moritz Mating, Soroush Sharbati, Ralf Einspanier

**Affiliations:** Institute of Veterinary Biochemistry, Freie Universität Berlin, Berlin, Germany

**Keywords:** *Apis mellifera*, *Varroa destructor*, formic acid, detoxification, 10-FTHFDH, apiculture, honey bee (*Apis mellifera* L.)

## Abstract

Honeybees are important managed pollinators that perform important ecological and economic functions. In recent decades, the obligate ectoparasite *Varroa destructor* severely affected survival of honeybees as it either feeds on hemolymph and fat bodies or acts as a vector for viruses. A common treatment against the varroa mite is formic acid, which has been used for many years by beekeepers. This treatment is known to be effective, but the therapeutic index is very narrow. Many beekeepers report negative effects of formic acid on bees, which include damage to brood, worker bee mortality, and queen loss. Little is yet known about the molecular mechanisms of formic acid detoxification in honeybees. Our previous study shows the upregulation of predicted 10-formyl tetrahydrofolate dehydrogenase (10-FTHFDH) transcripts in honeybees exposed to formic acid. Here, the predicted honeybee-specific 10-FTHFDH is recombinantly expressed, and its hydrolase and dehydrogenase activities are investigated. As a result, the enzyme shows similar dehydrogenase activity in comparison to known 10-FTHFDHs. This study provides further knowledge to better understand the detoxification mechanisms of formic acid in *Apis mellifera*.

## Introduction

Honeybees provide essential ecological and economic functions in our modern society. Honeybees are main pollinators for many agricultural and flowering plants in general (1, 2). Many plants are vastly reliant on pollination through bees, such as almonds, avocados, blueberries, onions, and many more (3). Overall, the economic value worldwide is estimated at up to 190 billion euros (4). For the last decades, a strong decline in honeybee populations is reported around the world. In the United States managed honeybee colonies declined from a peak in 1940 of around 6 million colonies to just over 2 million colonies in 2008 (5). This decline has remained constant over the last 10 years with an annual colony loss of about 40% (6). It is, therefore, predicted that one day the critical number may be reached at which there will no longer be enough bees and other pollinators (7). In addition to malpractices of beekeepers, increased use of pesticides by farmers, and the emergence and prevalence of pathogens, varroa mites are one of the major factors in the loss of colonies (8, 9). *Varroa destructor* (10) as an obligate ectoparasitic mite that feeds on the fat body and hemolymph of larvae and adult bees (11) directly weakens the larvae and imagos. In addition, varroa is known as a vector of various pathogens, including viruses, such as Deformed wing virus, Sacbrood virus, and Acute bee paralysis virus (12, 13) as well as bacterial pathogens like American foulbrood (14).

Early treatments against *V. destructor* are essential and comprise the usage of synthetic pyrethroids, such as Fluvalinate and amidines, such as Amitraz (8, 15). A common problem is the quick development of resistance, which also leads to cross-resistances against other pyrethroids (8, 16, 17). Additionally, due to the lipophilic character of the pyrethroids, residues of the chemicals can be found in bee products, such as wax and honey (18, 19). Alternatives to those synthetic chemical compounds are organic acids. Naturally occurring acids, such as oxalic, lactic, and formic acid, are licensed for application in varroa-infested hives in the EU, United States, and Canada as well as most of Latin America, including Argentina, Colombia, Costa Rica, and more (20–26). Formic acid has a very low risk of leaving residues in bee products compared with synthetic acaricides when used correctly (27, 28). The application of formic acid does not exclusively provide health benefits for honeybees. Many different factors influence the efficiency of the treatment, such as temperature, humidity, colony strength, and presence of larvae as well as type and position of used applicator (9, 21). Additionally, the therapeutic index is very narrow, which could lead to damaged larvae and juveniles. Even though formic acid has been used for many years to control varroa, the molecular mechanisms for detoxification are widely unknown in honeybees. Our recent data show that the mRNA of the enzyme cytosolic 10-formyl-tetrahydrofolate dehydrogenase is upregulated in honeybees treated with formic acid (29).

Tetrahydrofolate (THF) is an essential molecule involved in the universal one-carbon (1C) metabolism, including purine and thymidine synthesis and homocysteine remethylation. The term “folate” in general includes molecules with three chemical parts: a pteridine ring, a para-aminobenzoic acid (PABA), and a polyglutamate tail. The bioactive form of folate is called tetrahydrofolate (THF), which is the reduced form of folic acid. The formyl group can exist in three different carbon oxidation states, all of which have different biochemical functions: 5,10-methylene-THF, 5-methyl-THF, and 10-formyl-THF (30). 10-formyl-THF is the most oxidized naturally occurring folate carbon. It is required for the *de novo* synthesis of purines. In proliferating cells *in vitro*, purine synthesis is the largest demand for 1C units (30, 31). In bacteria as well as in mitochondria, initiator methionine tRNAs are formylated by a process using 10-formyl-THF (32). The most remarkable property of 10-formyl-THF for our studies is that the 1C unit can be completely oxidized to CO_2_ in an NADP^+^-dependent reaction, which could easily remove, for example, formic acid from the organism (33). As reported for mammals, the folate-dependent One-Carbon-Pool (C_1_) is the most important detoxification pathway of formic acid, catalyzing the conversion of tetrahydrofolate (THF) to 10-formyltetrahydrofolate (10-THF) by a 10-formyltetrahydrofolate synthase (34). Subsequently, the aforementioned 10-formyltetrahydrofolate dehydrogenase catalyzes the NADP^+^-dependent reaction of 10-THF to CO_2_ and THF (35, 36). Formic acid is assumed to be toxic due to the inhibitory effect on the mitochondrial cytochrome oxidase, therefore, causing histotoxic hypoxia and acidosis (37, 38). The toxicity to mammals is highest after inhalation (LD_50_ of 7.4mg/l/4h in rats), but only low-to-moderate toxicity is observed with 145 mg/kg intravenous application in mice. No significant impacts on reproductivity, carcinogenicity, and genotoxicity have been found so far (39) with an exception in an *in vitro* study comparing the developmental toxicity on mouse and rat embryos after exposure to formic acid, where several defects, including open anterior and posterior neuropore were reported (40). These severe negative effects could not be confirmed *in vivo*, where the application of formic acid over several generations in rats does not result in negative effects (39). With LD_50_ ratios of 267 μg/ml/48 h for *A. mellifera* and 9μg/ml/48 h for *V. destructor*, the difference in tolerance between these two species is obvious (41). It was hypothesized that the lower LD50 and, thus, higher toxicity in *V. destructor* compared to *A. mellifera* could be explained by the difference in morphology and body size. Because the surface area is much larger compared to the body mass in varroa, more formic acid would be absorbed through the body surface. Apart from this hypothesis, neither physiological nor biochemical and molecular studies have been performed so far to explain the higher toxicity (42).

The aim of this study is to demonstrate and characterize the predicted function of the recombinantly expressed enzyme 10-formyl-tetrahydrofolate dehydrogenase of *Apis mellifera*. We show that this newly found insect enzyme has similar activities to previously described mammalian enzymes and, therefore, may play a key role in the detoxification of formic acid in honeybees.

## Materials and Methods

### *Apis mellifera* Sampling

One-day-old worker bees were collected from the apiary of the Institute of Veterinary Biochemistry, Freie Universität Berlin, Berlin (52.42898 °N, 13.23762 °E) using one queen-right colony with *A. mellifera (carnica)* in the summer season 2020. Colonies were healthy, had enough food supply, and showed no symptoms of diseases or increased parasitism. Individuals were shock-frozen in liquid nitrogen and stored at −80°C until further use.

### RNA Extraction

RNA extraction was performed using the Quick-RNA™ Miniprep Kit (Zymo Research Europe GmbH, Freiburg, DE). Briefly, individuals were lysed in a lysing Matrix S (MP Biomedicals, Heidelberg, DE) containing 1 ml of lysis buffer using a BeadBlaster (Benchmark Scientific, Edison, USA). Tubes were then centrifuged at 12,000 × g at 4°C for 10 min. Supernatant was transferred into a clean microcentrifuge tube containing 1.5x volume 100% ethanol. The solution was then used according to manufacturer's protocol. RNA was eluted in a total volume of 40 μl ddH_2_O. Quantity and quality of total RNA was analyzed using an agilent RNA 6000 nano chip on a 2100 Bioanalyzer (Agilent Technologies, California,USA). Isolated RNA was stored at −80°C until use.

### First Strand cDNA-Synthesis

Protoscript® II Transcriptase (New England Biolabs, Inc., Ipswich, USA) was used according to manufacturer's protocol. Briefly, 1 μg DNA-free RNA was incubated with 1 μl d(T)23VN-Primer (50 μM) and 1 μl Random Primer Mix (50 μM) at 65°C for 5 min in a total volume of 8 μl. Thereafter, 12 μl of Protoscript Mastermix was added, and the sample was incubated at 42°C for 60 min and heat inactivated at 80°C for 5 min. cDNA was then diluted by addition of 80 μl ddH2O and stored at −20°C in adequate aliquots. To create a broad library, 5 μl of each sample was added to one microcentrifuge tube before freezing.

### Sequence Alignment and BlastX

Clustal Omega (43) was used for sequence alignment. *Homo sapiens, Rattus norvegicus, Pongo abllei*, and *Mus musculus* amino acid sequences were retrieved from uniprot.org (Accesion Nr: O75891, P28037, Q5RFM9, Q8R0Y6), and predicted amino acid sequences of *A. mellifera* was retrieved from NCBI (Accesion Nr.: XP_026298140.1). A protein similarity summary was generated based on the DNA sequence of *A. mellifera* THFDH using BlastX (NIH).

### Creation of pFBD-eGFP-Amel_10-FTHFDH Expression Vector

The open reading frame of the *A. mellifera* cytosolic 10-formyltetrahydrofolate dehydrogenase (Accession: XM_026442355.1) (Amel_10-FTHFDH) was amplified by polymerase chain reaction (PCR) using the primers Amel_FTHFDH_ORF_F/R (5′-ATGGCGCAACTCAAAGTGGC; 5′-CTAATATTCTACAGTGATAGTTTTTG). The PCR product was then subcloned into pJet1.2 vector (Thermo Scientific, Karlsruhe, DE) for sequencing and creation of a template for further use. The ORF-containing vector was used to create overhangs containing restriction sites (BamHI, NotI), and a 6x-HisTag at the N-terminus for later purification of the protein. pFastBacDual (pFBD) vector of the Bac-to-Bac System (Thermo Scientific, Karlsruhe, DE) with an enhanced green fluorescent protein (eGFP) cloned at the p10-promoter site was used as expression vector. The insert was created by PCR using the Amel_FTHFDH_BHI_HT_F and Amel_FTHFDH_NotI_R primers (5′-TCATACGGATCCATGCACCACCACCACCACCACGCGCAACTCAAAGTGGC; 5′-TCATACGCGGCCGCCTAATATTCTACAGTGATAGTTTTTG). pFBD-eGFP was digested with appropriate restriction enzymes, and the vector was dephosphorylated using an Antarctic Phosphatase (New England Biolabs, Inc., Ipswich, USA) to prevent relegation. PCR product was ligated with the vector using a T4-ligase (New England Biolabs, Inc., Ipswich, USA) using standard protocols.

### Creation of the Recombinant Bacmid

To create the recombinant Bacmid, Gibco™ Max Efficiency™ DH10Bac competent Cells (Thermo Scientific, Karlsruhe, DE) were transformed using 1 μg of pFBD-construct. Cells were thawed on ice, and 1 μg construct was added. The mixture was incubated for 30 min on ice, heat-shocked for 45 s at 42°C, and transferred back to ice for 2 min. Then, 900 μl S.O.C medium were added. Culture was incubated for 4 h at 37°C in a shaking incubator at 225 rpm. Cells were plated on LB-medium containing 50 μg/ml kanamycin, 7 μg/ml gentamicin, 10 μg/ml tetracycline, 500 μg/ml X-Gal, and 1 μM IPTG. Plates were incubated for 48 h at 37°C. White colonies were restreaked and Bacmid isolated using manufacturer's protocol.

### Creation of Baculovirus

To create the recombinant baculovirus, Sf21 insect cells were transfected with 1 μg Bacmid DNA, and 6 μl Gibco™ Cellfectin™ II reagent (Thermo Scientific, Karlsruhe, DE) was used as suggested by the manufacturer. Successful transfection was monitored by expression of eGFP under an inverse fluorescent microscope DMI 6000B (Leica), and photos were taken using a DFC 365FX (Leica) camera. Virus stock was extracted by detaching cells from the flask and centrifuging at 3,000 × g for 5 min. Virus-containing supernatant was transferred into a sterile 15 ml centrifuge tube and stored safe from light at 4°C until further use.

### Expression and Purification of Recombinant Protein

To produce recombinant protein, Hi5 cells at 80–90% confluency were used, and 3 × 10^6^ cells were seeded into a T175 flask (Sarstedt) containing 50 ml of Gibco™ ExpressFive™ SFM (Thermo). Next, 30 μl/ml virus stock was added, and cells were incubated at 27°C for 4 days or until most cells showed eGFP expression. Cells were pelleted at 5,000 × g for 20 min at 4°C. Pellets were resuspended in 20 ml PBS containing EDTA-free proteinase inhibitor cocktail (SIGMA). The suspension was sonified on ice using a sonifier 250 (Branson) for 4 min with an amplitude of 2 and at 20% energy. The suspension was cleared by centrifugation at 5,000 × g for 20 min at 4°C. Protein-containing supernatant as well as PBS containing Imidazole at different concentrations [10 mM (Equilibration Buffer), 25 mM (Wash Buffer), 100, 150, 200, and 500 mM (Elution Buffer)] were particle-free filtered (0.4 μM pore size, PES); 2 ml bed-volume (BV) of HisPur™ Ni-NTA Resin (Thermo) was equilibrated with 5 BV Equilibration buffer. Protein was equilibrated with 20 ml equilibration buffer and added to the column. The column was washed with 20 BV wash buffer, and thereafter four elution fractions were obtained using four different concentrations of imidazole (100, 150, 200, and 500 mM). The whole purification was performed at 4°C. Thereafter, to remove impurities and Imidazol from the enzyme, protein concentrators with a molecular weight cutoff of 50 kDa (Pierce) were used as suggested by the manufacturer.

### Synthesis of 10-Formyl Tetrahydrofolate

To synthesize the substrate 10-formyl tetrahydrofolate, an established protocol by Rabinowitz et al. (44) was used. Briefly, 100 mg of dl-5-formyltetrahydrofolic acid (SIGMA) were dissolved in 8 ml of 1 M β-mercapto-ethanol (Roth). The pH was adjusted to 1.5 with HCl. The mixture was stored at 4°C for at least 12 h. The solution, now containing dl-5,10-methenyltetrahydrofolic acid as a precipitate (bright yellow tint), was adjusted to a pH of 8 with KOH, purged with Argon, and incubated overnight at 4°C in an evacuated vessel. The solution now containing 10-formyl tetrahydrofolate (clear color) was directly used for assays.

### Enzyme Activity Assays

All assays were performed in a ClarioStar plus multimode-plate reader (BMG labtech); 100 mM β-mercapto-ethanol, 200 μM NADP^+^ and 10 μg of purified enzyme were added to each well and incubated at 30°C for 2 min. Substrate was injected using built-in injectors at different concentrations. NADPH production was monitored at 340 nm for a period of 30 min. All substances were diluted in Tris/HCl buffer (pH 6.8–8.4) to a total of 100 μl. Km and V_max_ have been calculated using a molar extinction coefficient of 6220 M^−1^cm^−1^ for NADPH. For the hydrolase activity assay, the abovementioned conditions were used, but NADP^+^ was omitted and production of the product was monitored at a wavelength of 300 nm.

### Analysis of Kinetic Data

Initial reaction rates were used to determine the respective enzyme activities. Kinetic parameters were derived by using GraphPad Prism version 9.0.2 (for Windows 10, GraphPad Software, San Diego, California USA, www.graphpad.com), which determines kinetic parameters from the Michaelis–Menten equation by using non-linear regression.

## Results

### Sequence Alignment and BlastX

**Figure 1:**
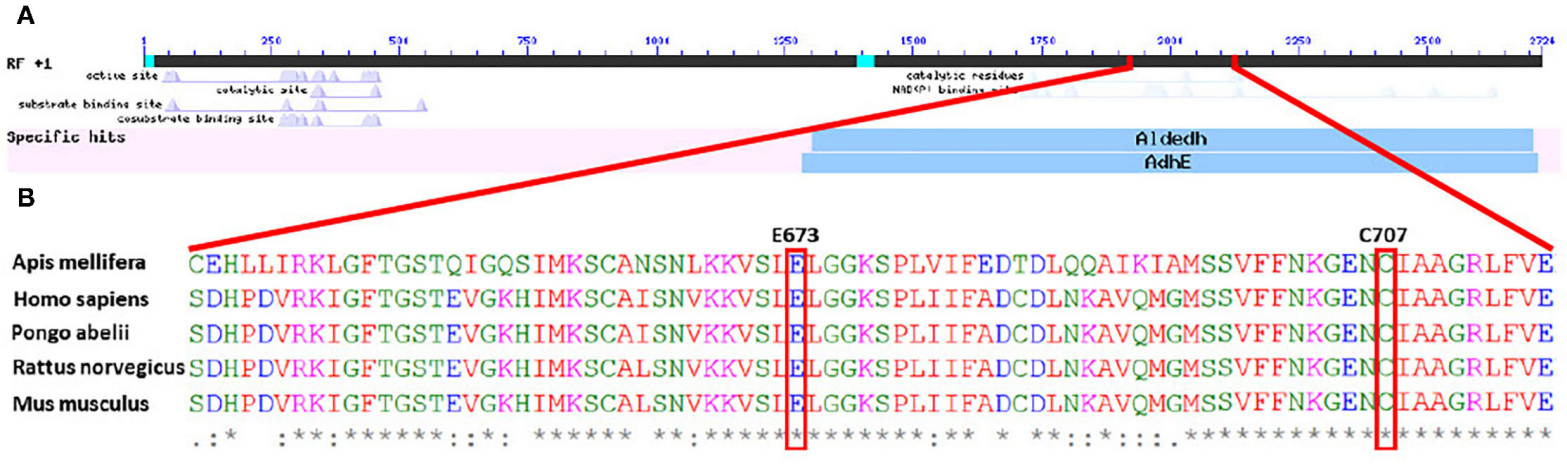
**(A)** Graphic overview of BlastX of the DNA sequence of Amel_FTHFDH, showing specific hits for known enzymes. The dehydrogenase subunit is identified as such, whereas the potential hydrolase subunit is not identified. **(B)** Partial amino acid sequence alignment of different 10-formyl tetrahydrofolate dehydrogenases. Highlighted amino acids are important residues in the active sites of mammalian dehydrogenases.

General sequence alignment of known 10-formyl tetrahydrofolate dehydrogenase (10-FTHFDH) sequences of *Homo sapiens, Rattus norvegicus, Pongo abelii*, and *Mus musculus* with the sequence of the *A. mellifera* 10-FTHFDH show a high percentage identity between mammals (ca. 91% homology). In contrast, the percentage homology between mammals and *A. mellifera* was at about 60% (Table 1). This value increased when only the specific sequence regions of the dehydrogenase domains were aligned (~70% homology). When only the hydrolase domains were aligned, homology values decreased to 55%. BlastX revealed that the dehydrogenase domain is recognized as such, but the hydrolase domain is no longer recognizable by the analysis (Figure 1A). Sequence alignment also revealed that important regions such as E673 and C707 of the dehydrogenase domain, known to be key residues in the active site, are highly conserved (Figure 1B).

**Table 1:** Percentage identiy matrix, showing overall percentage identity between total 10-FTHFDH (T), Hydrolase domain of 10-FTHFDH (H), and dehydrogenase domain of 10-FTHFDH (D).

		***Apis mellifera***	**Homo sapiens**	**Pongo abelii**	**Rattus norvegicus**	**Mus musculus**
	T	100				
*Apis mellifera*	H	100				
	D	100				
	T	59.71	100			
*Homo sapiens*	H	55.39	100			
	D	69.65	100			
	T	59.71	98.34	100		
*Pongo abelii*	H	55.02	98.52	100		
	D	69.85	94.19	100		
	T	60.04	91.80	92.02	100	
*Rattus norvegicus*	H	56.51	93.70	93.70	100	
	D	69.85	92.95	93.36	100	
	T	60.04	92.35	92.68	97.67	100
*Mus musculus*	H	56.13	93.70	93.70	97.78	100
	D	70.06	94.19	94.61	98.34	100

*Within mammals, the percentage identity is above 91% in all categories, whereas the percentage identity between total A. mellifera 10-FTHFDH and mammals is at about 60% with an increase in percentage identity in the dehydrogenase domain and a decrease in the hydrolase domain, with around 70 and 55%, respectively*.

### Amel_10-FTFDH Expression and Purification

**Figure 2:**
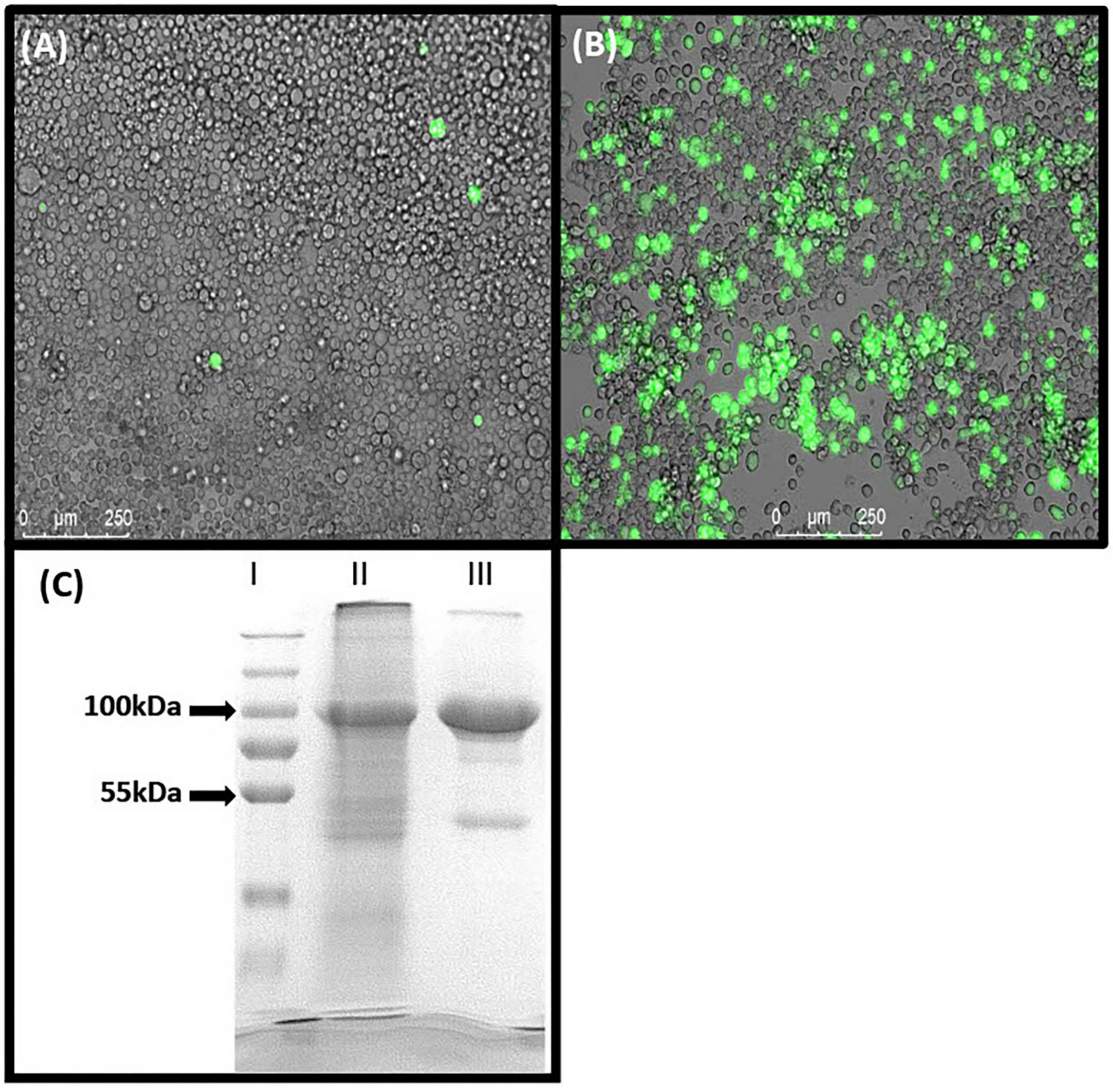
Protein expression. **(A)** SF21 cells expressing eGFP after transfection with Bacmid DNA. **(B)** Hi5 cells expressing eGFP 1 day before harvesting. **(C)** SDS-PAGE of (I) Marker, (II) Lysate of Hi5 Insect cells overexpressing Amel_FTHFDH and (III) purified Amel_FTHFDH. Proposed specifically expressed recombinant protein appears at 100 kDa.

10-formyl-tetrahydrofolate dehydrogenase of *A. mellifera* was successfully expressed and purified, containing a N-terminal 6x-HisTag, and 7 × 10^7^ Hi5-Insect cells resulted in a total yield of ~2 mg purified protein. Instead of using classical viral plaque assays, the expression of eGFP was used as a measure for a successful target protein production (Figures 2A,B). The protein identification by means of SDS-PAGE revealed successful expression and purification of 10-FTFDH (Figure 2C). The calculated size of the predicted protein is expected to appear at 100 kDa, which was verified by the gel. The non-specific weak band at 52 kDa could probably be a degradation product.

### Enzymatic Activity and pH Dependence

**Figure 3:**
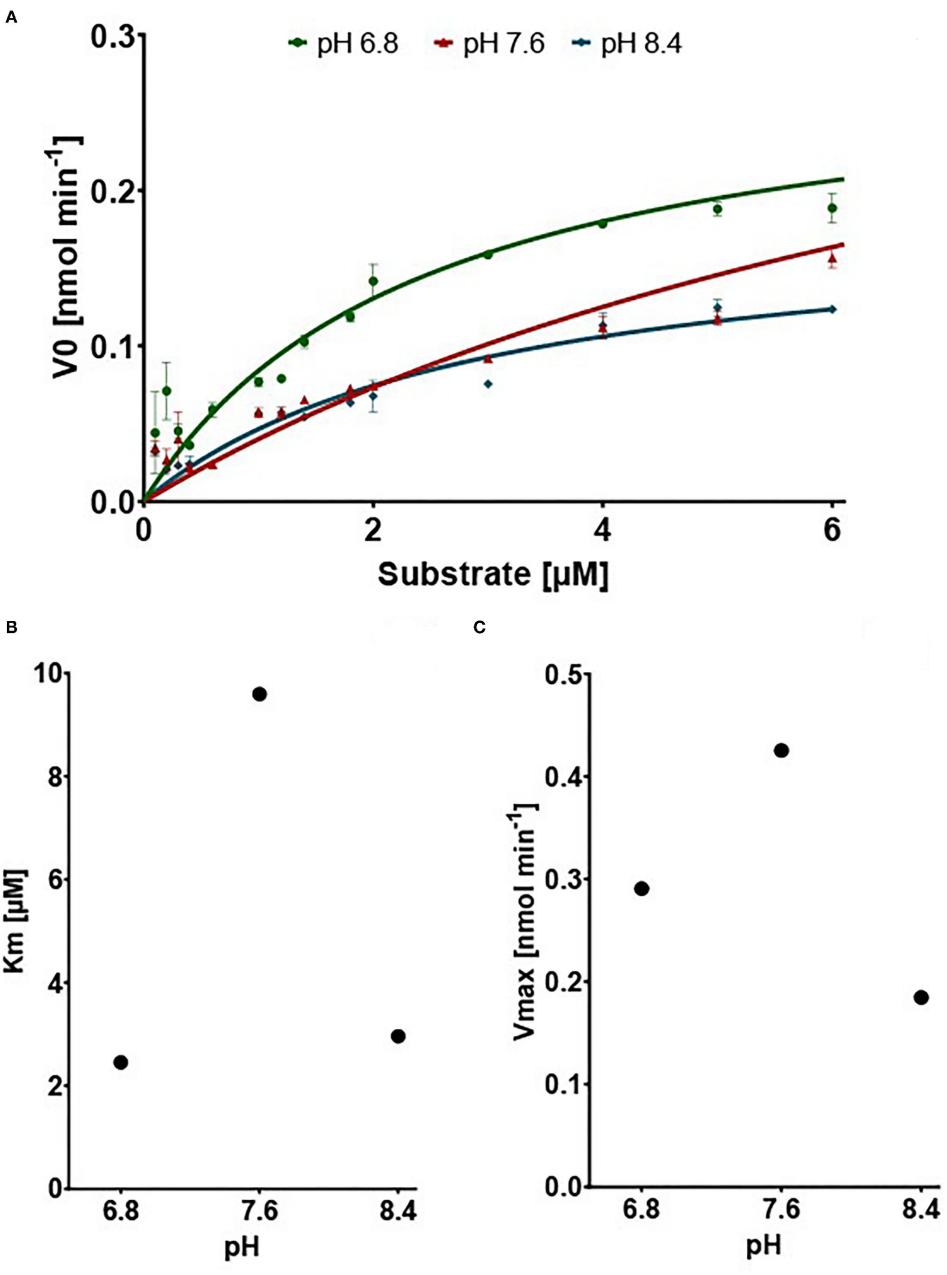
Results of the dehydrogenase assays. **(A)** V_0_ in nM min^−1^ in connection with increasing Substrate concentration. Points show mean of V0 replicates (n = 3) ±SEM **(B)** Vmax in nmol min^−1^ dependent on pH **(C)** Km in μM dependent on pH.

We show that the expressed 10-formyl tetrahydrofolate dehydrogenase of *A. mellifera* shows dehydrogenase activity but does not show any hydrolase activity (SI1). The dehydrogenase activity was measured by monitoring the increase of absorbance at 340 nm, the maximum extinction peak of NADPH. Typical Michaelis–Menten kinetics are observed at all tested pH values (Figure 3A). The affinity of the enzyme for its substrate is indicated by its Km value. As depicted, the Km value is dependent on the pH with its optimum, indicated by low Km value, at pH 6.8 with a value of 2.455 μM. At pH 7.6 and 8.4, the Km increases to 9.596 μM and 2.961 μM, respectively (Figure 3B). The enzyme shows an optimal activity, which is indicated by a high Vmax, at pH 7.6 with a value of 0.4253 nM min^−1^. At the pH of 6.8 and 8.4, the enzyme expresses an activity of 0.2907 nM min^−1^ and 0.1846 nM min^−1^, respectively (Figure 3C).

## Discussion

This study shows for the first time that the predicted 10-formyl tetrahydrofolate dehydrogenase (10-FTHFDH) of *A. mellifera* (XM_026442355.1) can be expressed *in vitro*, and the resulting enzyme exhibits the expected dehydrogenase activity for the substrate 10-formyl tetrahydrofolate. In insects, there are to date no publications on the formic acid detoxifying enzyme 10-FTHFDH; in contrast, mammalian 10-FTHFDHs have been well-studied, particularly those of rat, mouse, and human (45–48).

Our study demonstrates an optimum of enzymatic activity at a neutral to basic pH. Similar results were previously reported for a recombinantly expressed 10-FTHFDH of *Rattus norvegicus* (49). The study reports the V_max_ value of about 0.095 μmol min^−1^ mg^−1^. In comparison, using the same units, we report a V_max_ of 0.043 μmol min^−1^ mg^−1^ of enzyme. The K_m_ value usually depends on the pH at which the reaction takes place. In our case it has a maximum at a pH of 7.6. The Km value in our study, 9.6 μM, is in a similar range to that of rat, 5.5 μM, and pig, 7.5 μM (49, 50). In both studies, the maximum activity was at neutral to basic pH (7.6–7.7), which supports our findings. Thus, the activity of the enzyme 10-FTHFDH, characterized for the first time in an insect, is comparable to the known activity values for the previously described representatives from mammals.

All 10-FTHFDH described so far are divided into two domains, comprising (I) a hydrolase domain, which catalyzes the NADP^+^-independent reaction of 10-formyltetrahydrofolate to tetrahydrofolate and formate and (II) a dehydrogenase domain, which catalyzes the NADP^+^-dependent reaction of 10-formyl tetrahydrofolate to CO_2_, tetrahydrofolate and water (45, 49). Tsybovsky and Krupenko (51) propose the following mechanism for the dehydrogenase catalysis. Glutamate E673 is hydrogen bonded to cysteine C707. The binding of NADP+ results in the rotation of the glutamate sidechain away from the cysteine, which simultaneously loses a proton; thereafter, the negatively charged sulfur of the cysteine forms a transient covalent bond with the C4 atom of the nicotinamide ring of the coenzyme. In the two phases of the dehydrogenase catalysis, acetylation and deacetylation, the cysteine functions as a catalytic nucleophile, whereas the glutamate is postulated to activate a water molecule in the deacetylation step. With the proposed mechanism, the two mentioned residues are of great importance. The amino acid sequence alignment showed a high percentage identity within the group of mammals analyzed, whereas the comparison of our honeybee 10-FTHFDH protein to the mammal enzymes shows a way lower percentage identity of about 60%. However, looking more closely at the specific domains, the dehydrogenase domain shows a marked increase in amino acid sequence homologies (from 60% to about 70%). Especially the previously mentioned functionally important residues and the regions in the surrounding area are highly conserved (Figure 1B). However, with an overall percentage identity of 70%, further studies should be performed to verify the active site. The hydrolase domain, on the other hand, has a lower percentage protein homology (about 55%), which could explain the loss of hydrolase function.

Formic acid toxicity is directly related to a burst of reactive oxygen species and oxidative damage in cells induced by formic acid (52). In contrast, folate plays an important role in reducing this oxidative stress (53), which would likely be explained by an increase in detoxification capacity. In humans, folate coenzymes are known to play a vital role in cellular homeostasis. Animals in general cannot synthesize folate *de novo* and need to ingest folate through their diet. Insufficient folate uptake can lead to deregulation of methylation processes (54), increased fragility of chromosomes due to decreased DNA repair capabilities (55, 56), and altered protein expression (57). If we assume that folate supplementation increases the detoxification capacity of 10-formyl tetrahydrofolate dehydrogenase, folic acid supplementation in the diet of honeybees could be used to increase the desired detoxification of formic acid.

In summary, we could show for the first time that recombinantly expressed enzyme 10-formyl tetrahydrofolate dehydrogenase of *A. mellifera* exhibits comparable activity to similar enzymes described in mammals with a Vmax value of 0.4253 nM min^−1^ at optimal pH. The confirmed activity of this specific enzyme implies a critical role in the detoxification of formic acid in the honeybee. In the future, better knowledge of this detoxicating enzyme may support honeybees' tolerance to the widely used formic acid for the treatment of varroa mites.

## Data Availability Statement

The original contributions presented in the study are included in the article/supplementary material, further inquiries can be directed to the corresponding authors.

## Author Contributions

MM designed the study, performed experiments, analyzed data, and wrote first draft of the manuscript. SS provided advice in study design and revised and edited the manuscript. RE provided advice in study design, revised and edited the manuscript, supervised planning, and execution. All authors read and approved the final version of the manuscript.

## Conflict of Interest

The authors declare that the research was conducted in the absence of any commercial or financial relationships that could be construed as a potential conflict of interest.

## Publisher's Note

All claims expressed in this article are solely those of the authors and do not necessarily represent those of their affiliated organizations, or those of the publisher, the editors and the reviewers. Any product that may be evaluated in this article, or claim that may be made by its manufacturer, is not guaranteed or endorsed by the publisher.
